# Supplementary material for: A Humanized Yeast Phenomic Model of Deoxycytidine Kinase to Predict Genetic Buffering of Nucleoside Analog Cytotoxicity
Source: Genes (Basel). 2019 Sep 30;10(10):770. doi: 10.3390/genes10100770 (PMC6826991; doi:10.3390/genes10100770)

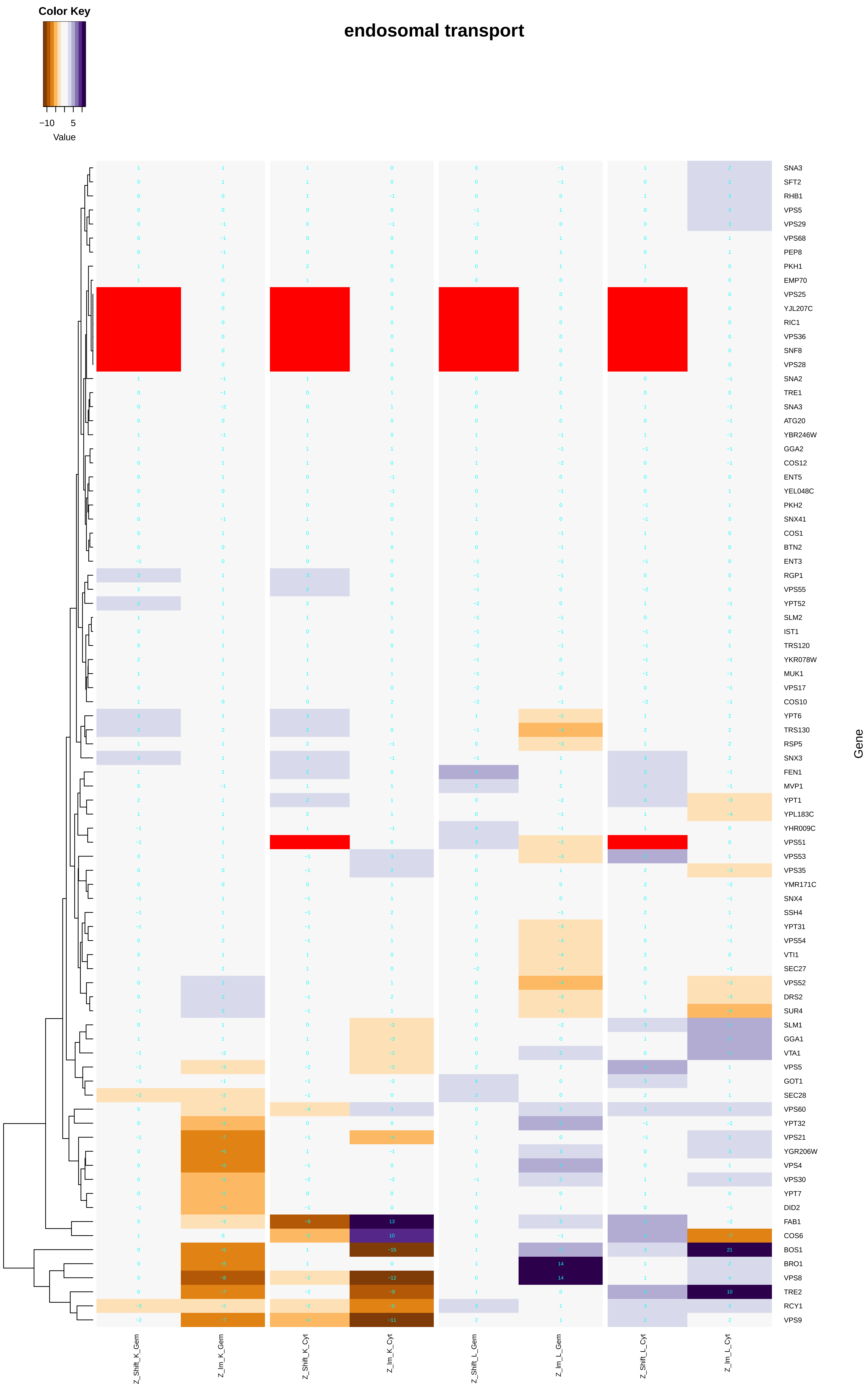

endosomal transport
Color Key
−10
5
Value
SNA3
SFT2
RHB1
VPS5
VPS29
VPS68
PEP8
PKH1
EMP70
VPS25
YJL207C
RIC1
VPS36
SNF8
VPS28
SNA2
TRE1
SNA3
ATG20
YBR246W
GGA2
COS12
ENT5
YEL048C
PKH2
SNX41
COS1
BTN2
ENT3
RGP1
VPS55
YPT52
SLM2
IST1
TRS120
YKR078W
MUK1
VPS17
COS10
YPT6
TRS130
RSP5
SNX3
FEN1
MVP1
YPT1
YPL183C
YHR009C
VPS51
VPS53
VPS35
YMR171C
SNX4
SSH4
YPT31
VPS54
VTI1
SEC27
VPS52
DRS2
SUR4
SLM1
GGA1
VTA1
VPS5
GOT1
SEC28
VPS60
YPT32
VPS21
YGR206W
VPS4
VPS30
YPT7
DID2
FAB1
COS6
BOS1
BRO1
VPS8
TRE2
RCY1
VPS9
Gene
Z_Shift_K_Gem
Z_Im_K_Gem
Z_Shift_K_Cyt
Z_Im_K_Cyt
Z_Shift_L_Gem
Z_Im_L_Gem
Z_Shift_L_Cyt
Z_Im_L_Cyt

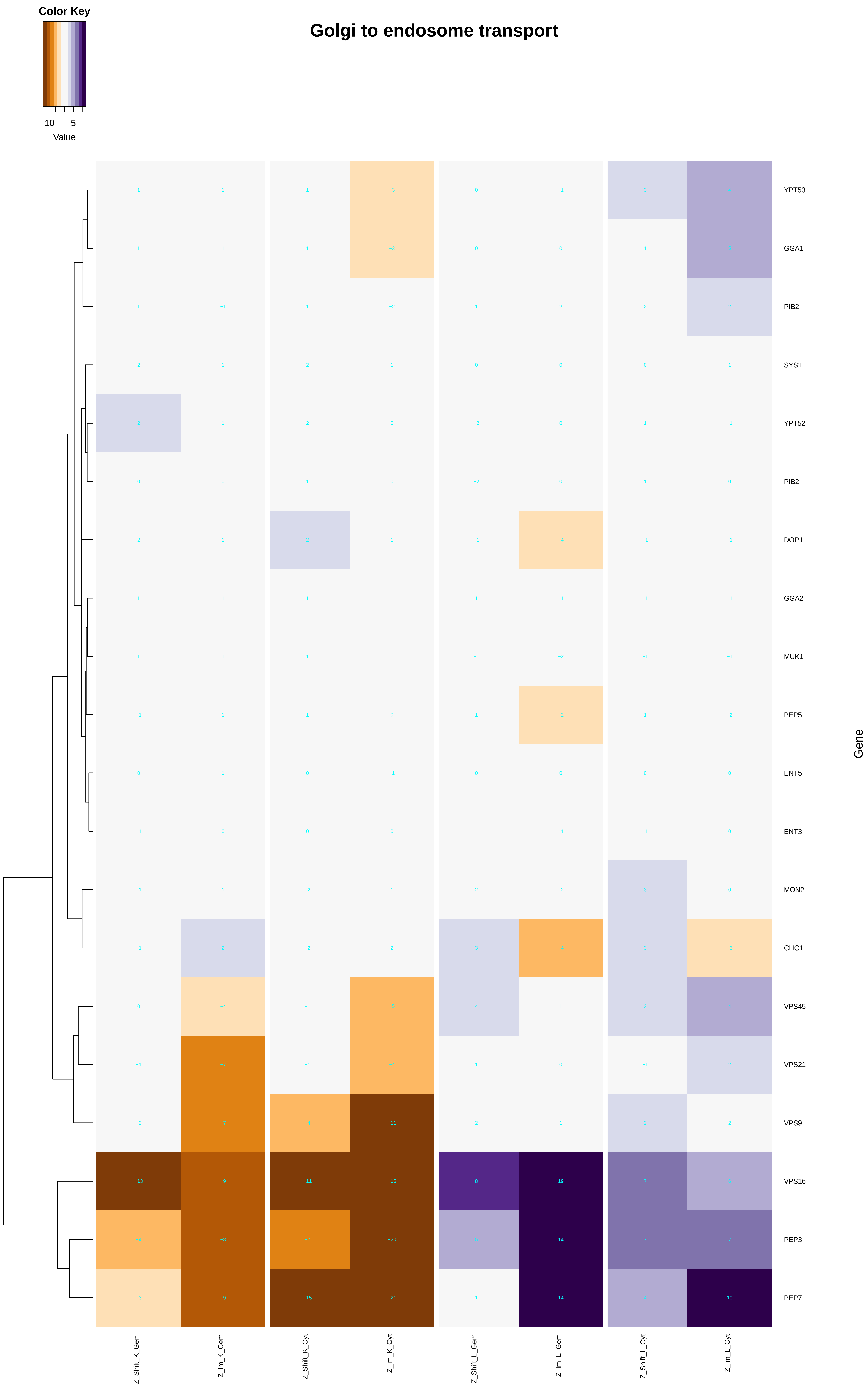
Color Key
−10
5
Value
Golgi to endosome transport
YPT53
GGA1
PIB2
SYS1
YPT52
PIB2
DOP1
GGA2
MUK1
PEP5
ENT5
ENT3
MON2
CHC1
VPS45
VPS21
VPS9
VPS16
PEP3
PEP7
Gene
Z_Shift_K_Gem
Z_Im_K_Gem
Z_Shift_K_Cyt
Z_Im_K_Cyt
Z_Shift_L_Gem
Z_Im_L_Gem
Z_Shift_L_Cyt
Z_Im_L_Cyt

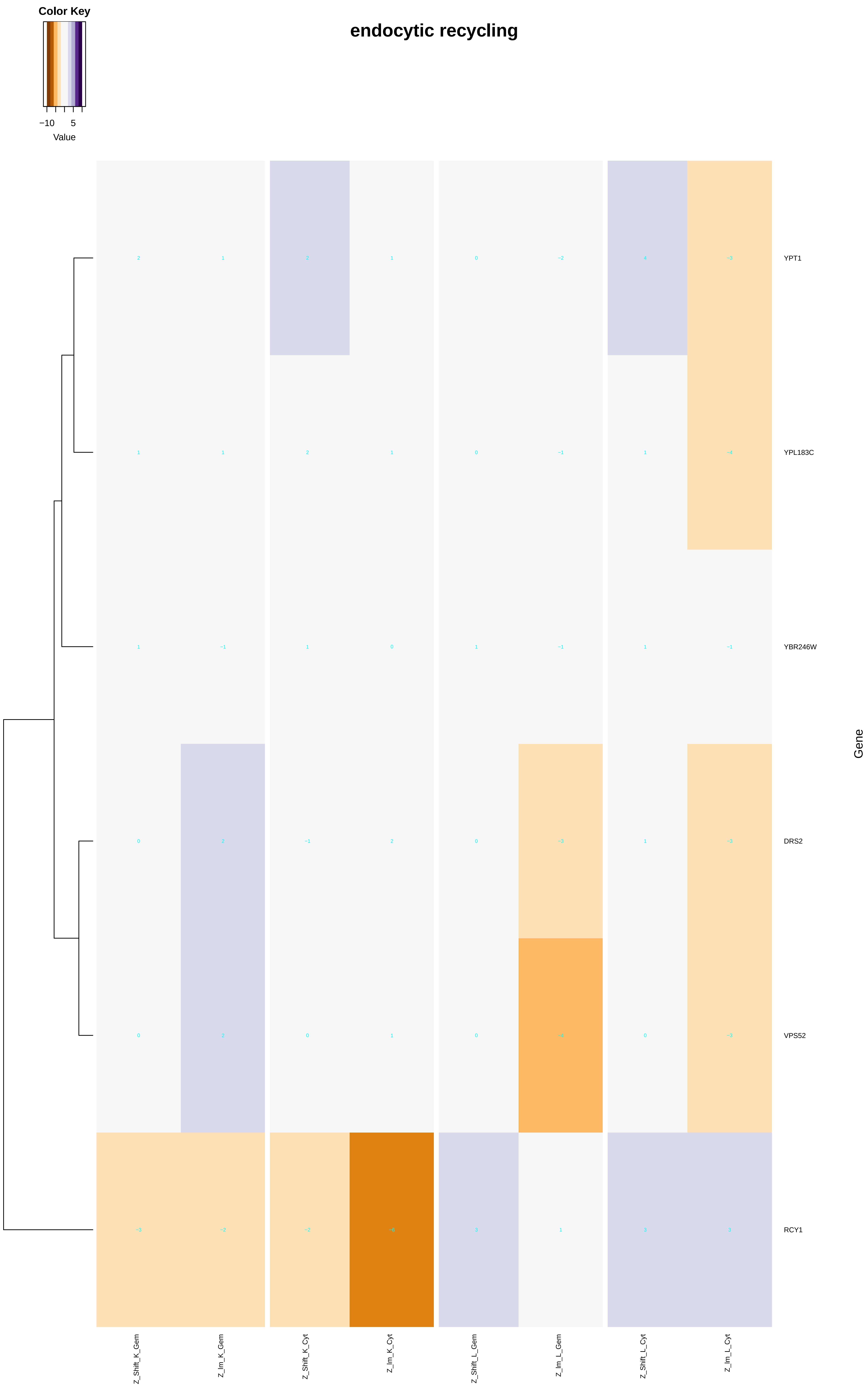

endocytic recycling
Color Key
−10
5
Value
YPT1
YPL183C
YBR246W
DRS2
VPS52
RCY1
Gene
Z_Shift_K_Gem
Z_Im_K_Gem
Z_Shift_K_Cyt
Z_Im_K_Cyt
Z_Shift_L_Gem
Z_Im_L_Gem
Z_Shift_L_Cyt
Z_Im_L_Cyt

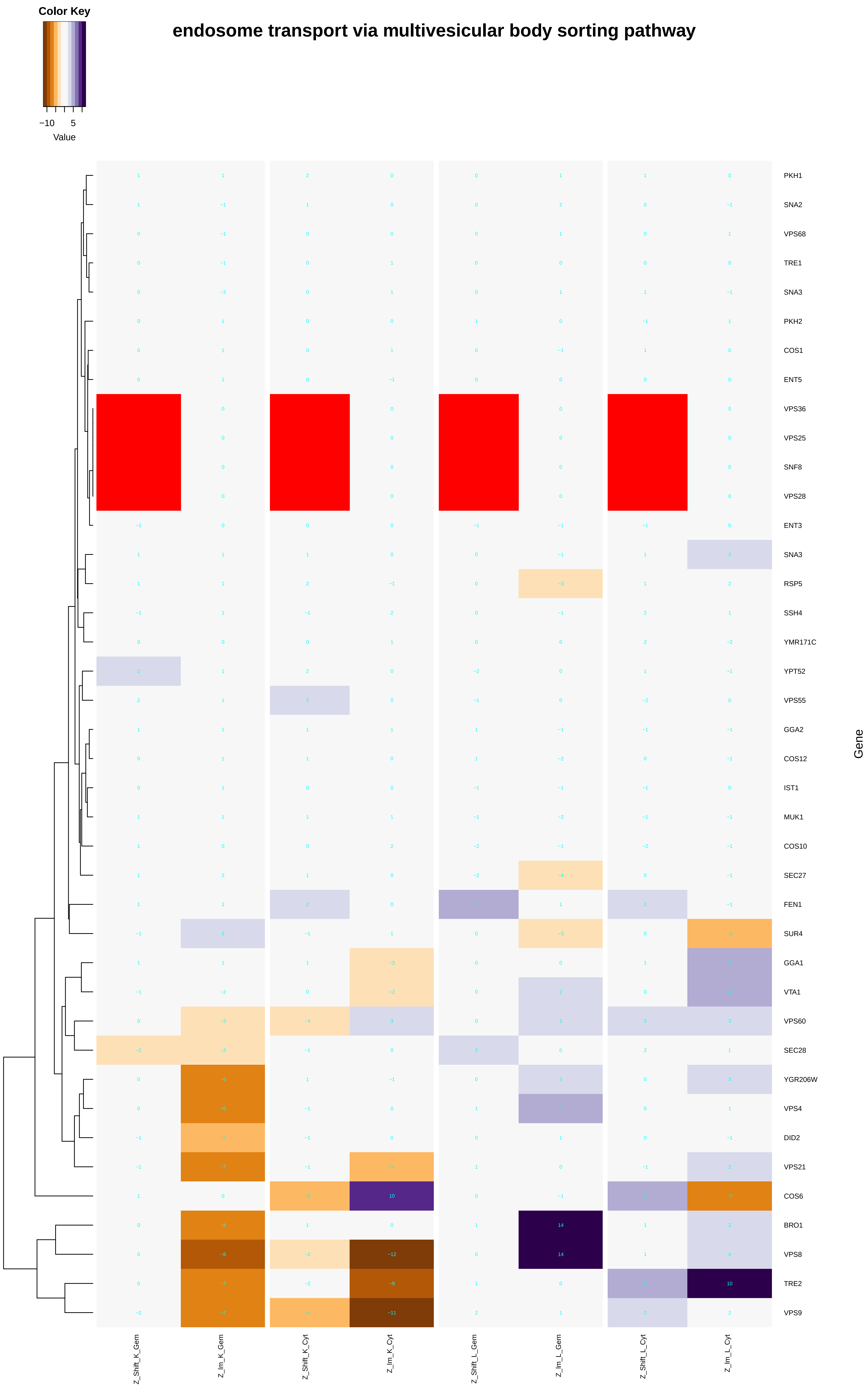

endosome transport via multivesicular body sorting pathway
Color Key
−10
5
Value
PKH1
SNA2
VPS68
TRE1
SNA3
PKH2
COS1
ENT5
VPS36
VPS25
SNF8
VPS28
ENT3
SNA3
RSP5
SSH4
YMR171C
YPT52
VPS55
GGA2
COS12
IST1
MUK1
COS10
SEC27
FEN1
SUR4
GGA1
VTA1
VPS60
SEC28
YGR206W
VPS4
DID2
VPS21
COS6
BRO1
VPS8
TRE2
VPS9
Gene
Z_Shift_K_Gem
Z_Im_K_Gem
Z_Shift_K_Cyt
Z_Im_K_Cyt
Z_Shift_L_Gem
Z_Im_L_Gem
Z_Shift_L_Cyt
Z_Im_L_Cyt

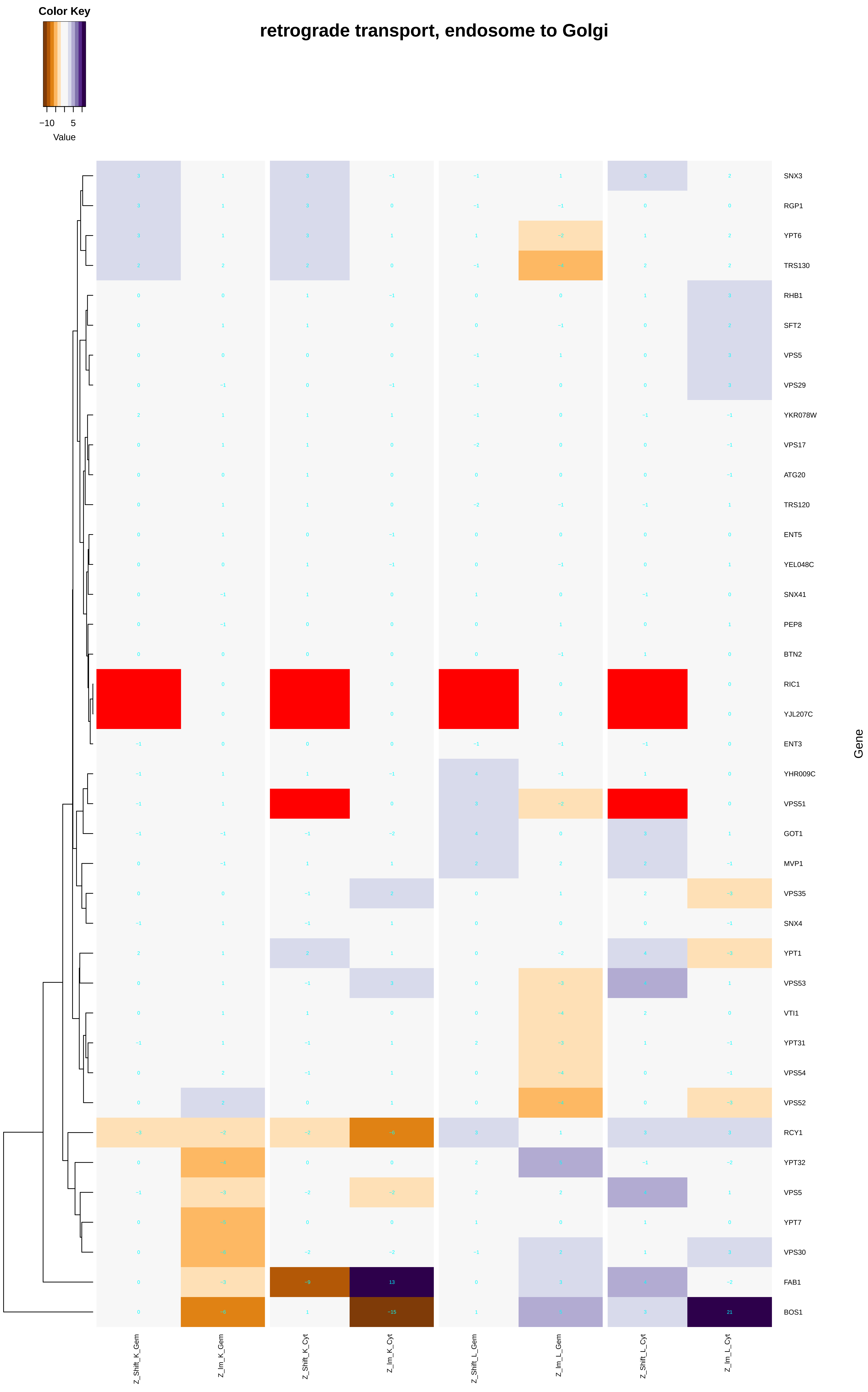
Color Key
−10
5
Value
retrograde transport, endosome to Golgi
SNX3
RGP1
YPT6
TRS130
RHB1
SFT2
VPS5
VPS29
YKR078W
VPS17
ATG20
TRS120
ENT5
YEL048C
SNX41
PEP8
BTN2
RIC1
YJL207C
ENT3
YHR009C
VPS51
GOT1
MVP1
VPS35
SNX4
YPT1
VPS53
VTI1
YPT31
VPS54
VPS52
RCY1
YPT32
VPS5
YPT7
VPS30
FAB1
BOS1
Gene
Z_Shift_K_Gem
Z_Im_K_Gem
Z_Shift_K_Cyt
Z_Im_K_Cyt
Z_Shift_L_Gem
Z_Im_L_Gem
Z_Shift_L_Cyt
Z_Im_L_Cyt

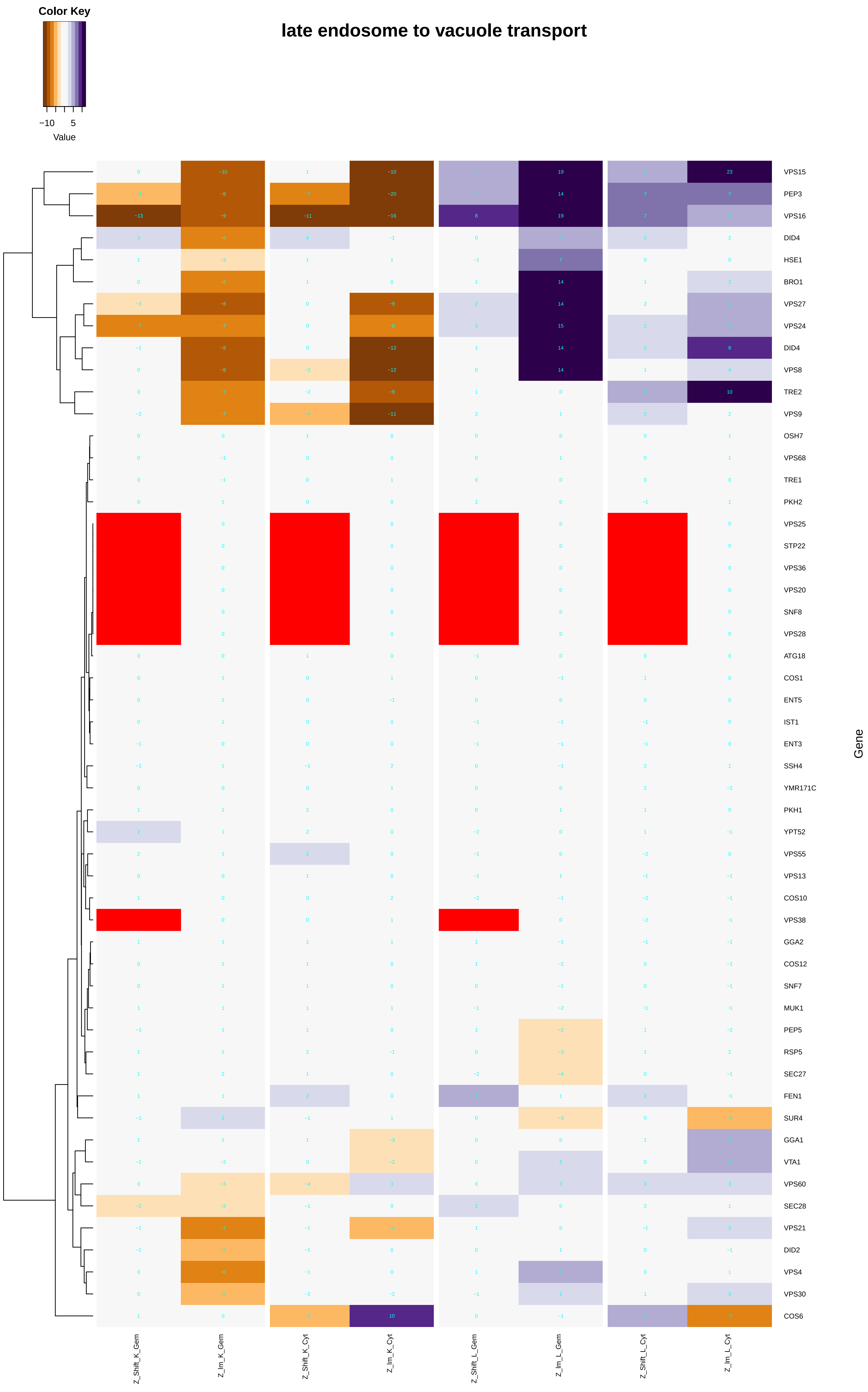

Color Key
−10 5
Value
late endosome to vacuole transport
VPS15
PEP3
VPS16
DID4
HSE1
BRO1
VPS27
VPS24
DID4
VPS8
TRE2
VPS9
OSH7
VPS68
TRE1
PKH2
VPS25
STP22
VPS36
VPS20
SNF8
VPS28
ATG18
COS1
ENT5
IST1
ENT3
SSH4
YMR171C
PKH1
YPT52
VPS55
VPS13
COS10
VPS38
GGA2
COS12
SNF7
MUK1
PEP5
RSP5
SEC27
FEN1
SUR4
GGA1
VTA1
VPS60
SEC28
VPS21
DID2
VPS4
VPS30
COS6
Gene
Z_Shift_K_Gem
Z_Im_K_Gem
Z_Shift_K_Cyt
Z_Im_K_Cyt
Z_Shift_L_Gem
Z_Im_L_Gem
Z_Shift_L_Cyt
Z_Im_L_Cyt

late endosome to vacuole transport via multivesicular body sorting pathway

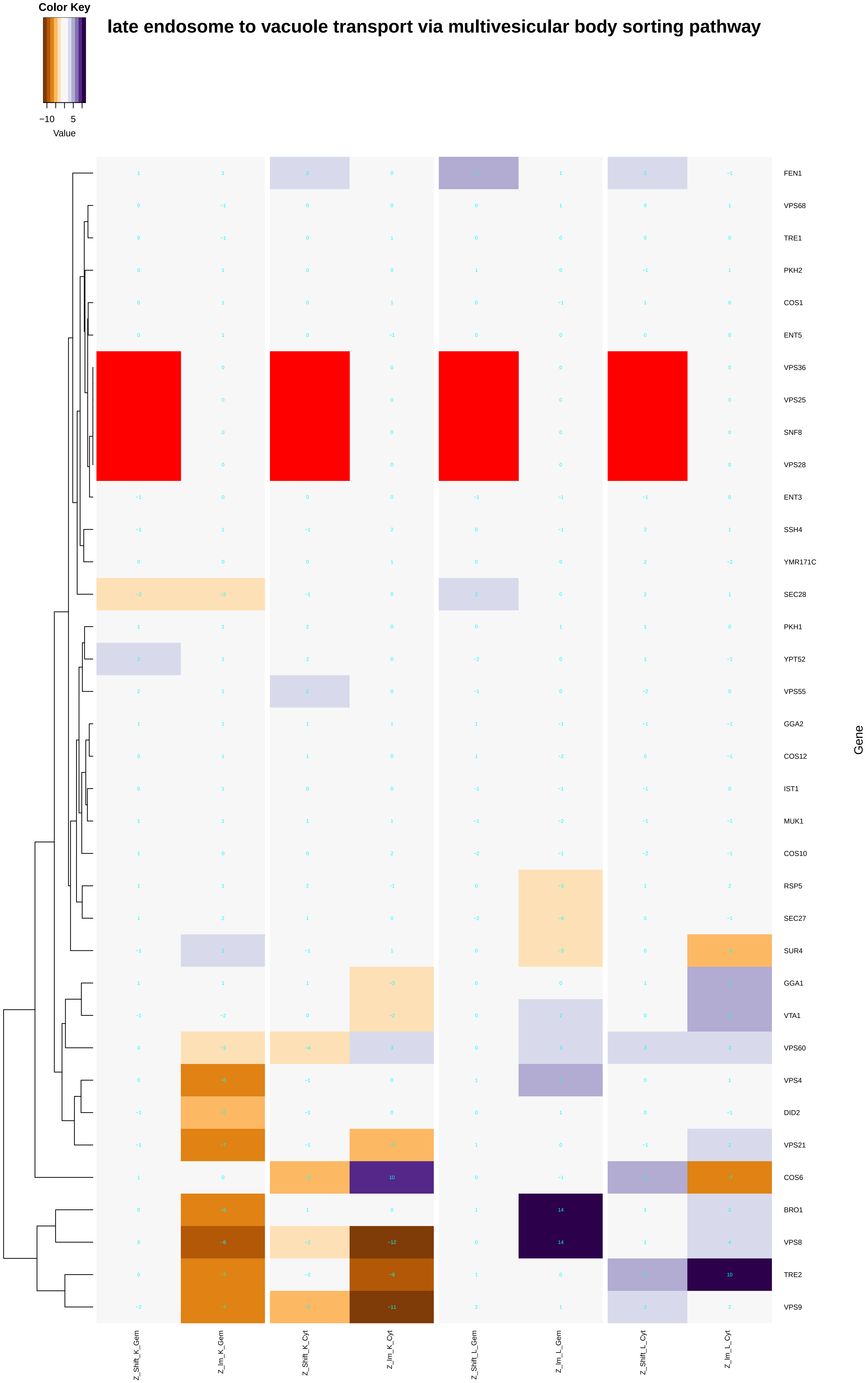

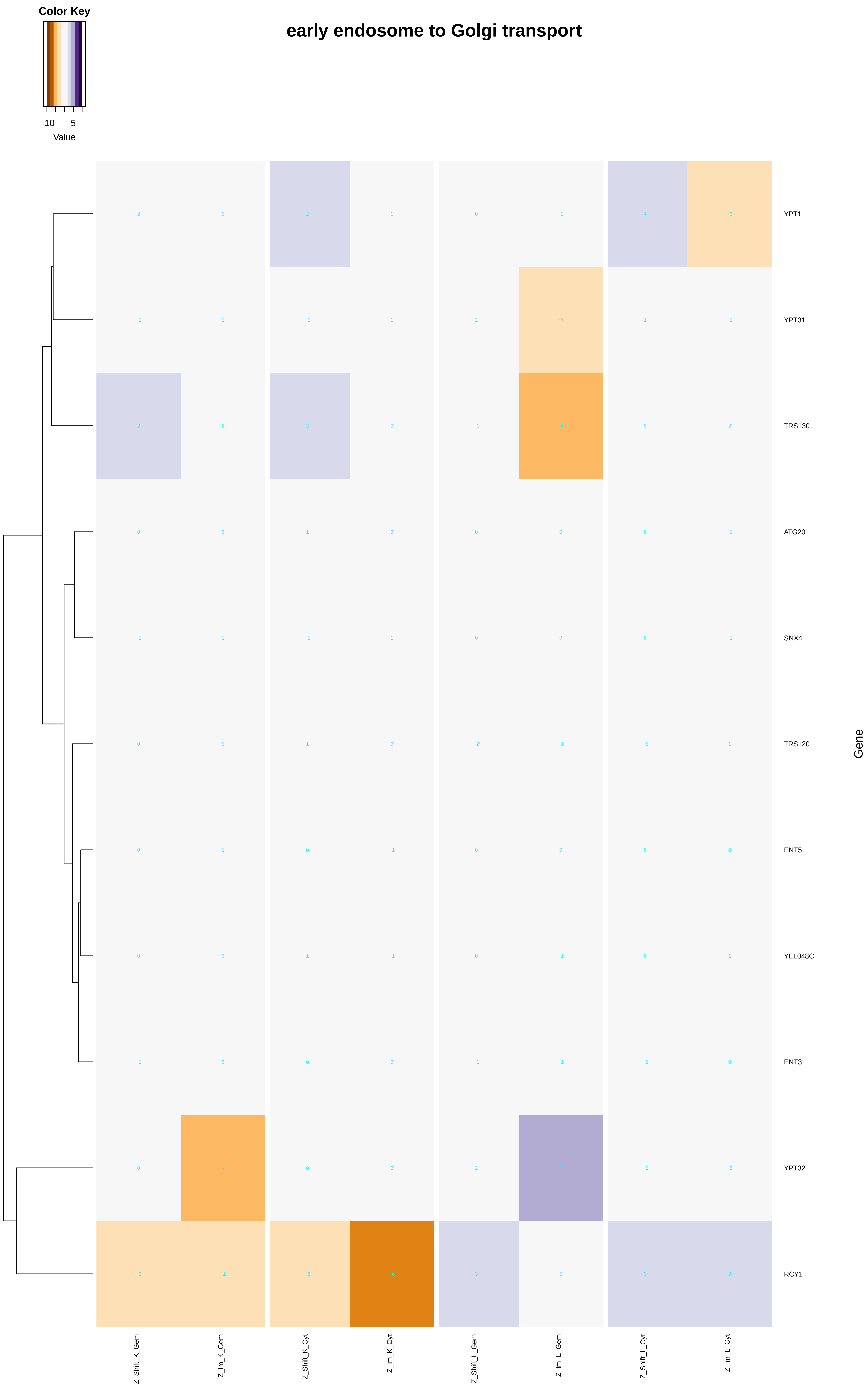

Color Key
−10
5
Value
early endosome to Golgi transport
YPT1
YPT31
TRS130
ATG20
SNX4
TRS120
ENT5
YEL048C
ENT3
YPT32
RCY1
Gene
Z_Shift_K_Gem
Z_Im_K_Gem
Z_Shift_K_Cyt
Z_Im_K_Cyt
Z_Shift_L_Gem
Z_Im_L_Gem
Z_Shift_L_Cyt
Z_Im_L_Cyt

le involved in ubiquitin−dependent protein catabolic process via the multivesicular body s

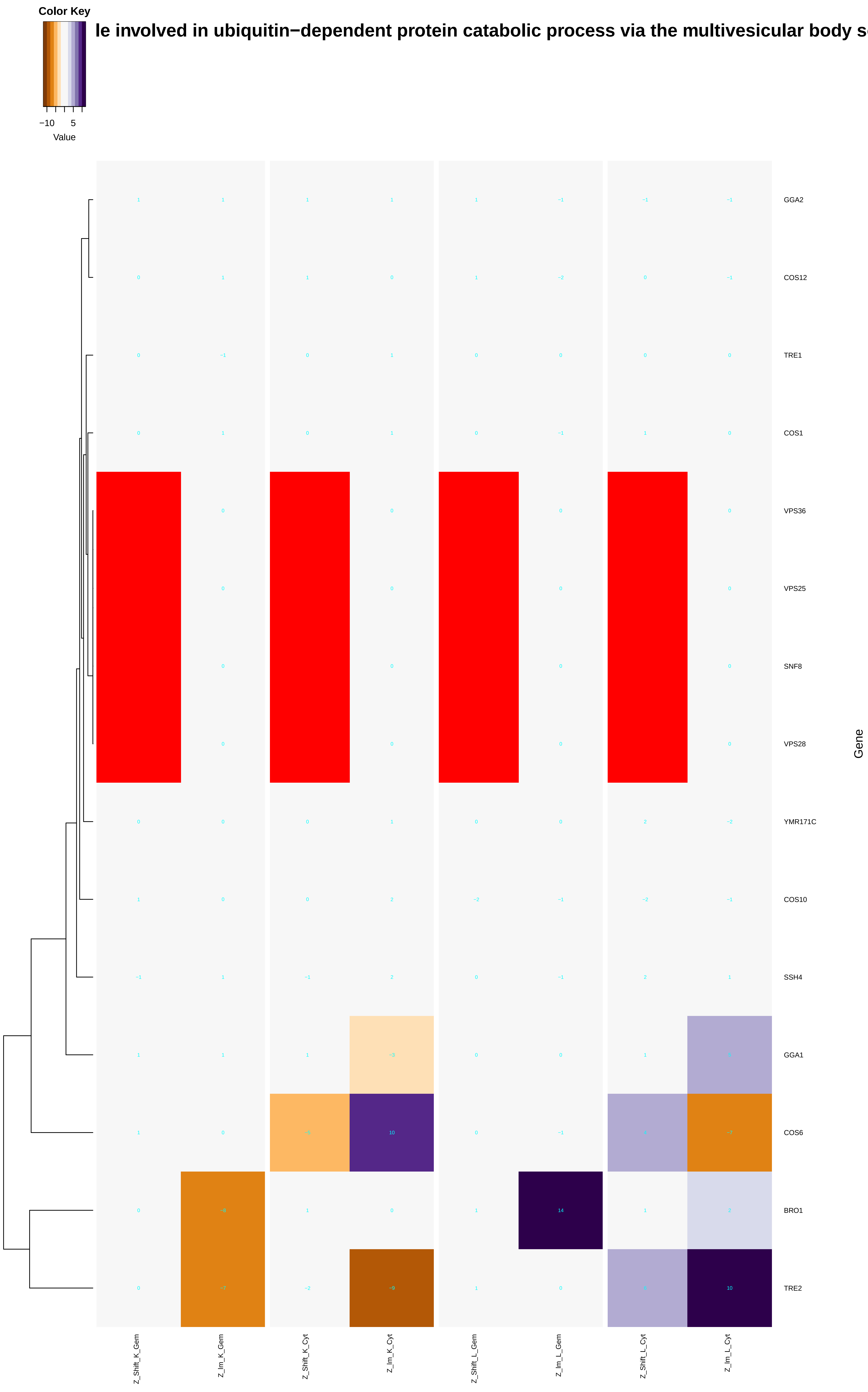

Supplement: Supplementary file 1 [file genes-10-00770-s001.zip › Additional_Files/Additional_File7_GOTermHeatmaps.zip!/Additional_File7_GOTermHeatmaps/endosomal_transport.pdf]
